# Characterisation of Structure-Borne Sound Source Using Reception Plate Method

**DOI:** 10.1155/2013/742853

**Published:** 2013-11-07

**Authors:** A. Putra, N. F. Saari, H. Bakri, R. Ramlan, R. M. Dan

**Affiliations:** Sustainable Maintenance Engineering Research Group, Faculty of Mechanical Engineering, Universiti Teknikal Malaysia Melaka, Hang Tuah Jaya, Melaka, 76100 Durian Tunggal, Malaysia

## Abstract

A laboratory-based experiment procedure of reception plate method for structure-borne sound source characterisation is reported in this paper. The method uses the assumption that the input power from the source installed on the plate is equal to the power dissipated by the plate. In this experiment, rectangular plates having high and low mobility relative to that of the source were used as the reception plates and a small electric fan motor was acting as the structure-borne source. The data representing the source characteristics, namely, the free velocity and the source mobility, were obtained and compared with those from direct measurement. Assumptions and constraints employing this method are discussed.

## 1. Introduction

 The structure-borne sound is still a challenging problem in engineering especially in buildings where machineries such as fans, compressors, hydraulic equipment, electrical motors, heating pumps, washing machines, and air conditioning system can produce a considerable amount of vibration [1]. The transmitted vibration waves do not only cause noise but also are hazardous to the building structure. Such machines are called structure-borne sound sources.

The symptom before the structural damage due to the effect of vibration is sometimes not visible. With the information of the vibration level strength of the structure-borne sound source, a preliminary control measure can be planned. This is where characterisation of the source becomes important [2]. Unfortunately, determination of the “behaviour” of the structure-borne source is more difficult compared to airborne source because the machine's vibration energy transmits to the supporting structure in a complicated motion [3]. It is significant to know as much information as possible not only about the source but also about the receiver structure to obtain the dynamic characteristics through the contact points represented by the mobility, that is, the ratio of the response velocity to the excitation force.

For structure-borne source characterisation, the reception plate method as a laboratory measurement test has been proposed [4, 5]. The vibration source is installed on the reception plate where it is assumed that the injected power by the source is equal to the power dissipated by the plate. By employing reception plates having mobility much greater or much lower than that of the source to enforce simplification in the mathematical model, from here, the free velocity of the source as well as the source mobility can be obtained [6]. However, using the plate power equation [7] in the reception plate method requires diffuse field vibration in the reception plate where the modal density should be sufficiently high. This is convenient for thin and high mobility plate, but problematic for the thick and low mobility plate.

In this paper, the reception plate method is again addressed and discussed. The methodology is similar to that in [6] where here, a small motor from a table fan was used as the structure-borne source. The damping of the reception plate was determined also from the plate power equation. It is shown that for the thick, low mobility reception plate, spatially averaged squared velocity can only be performed around the contact points where the near-field is dominant to obtain a better prediction of the source mobility.

## 2. Mathematical Formulation

### 2.1. General Formulation

**Figure 1:**
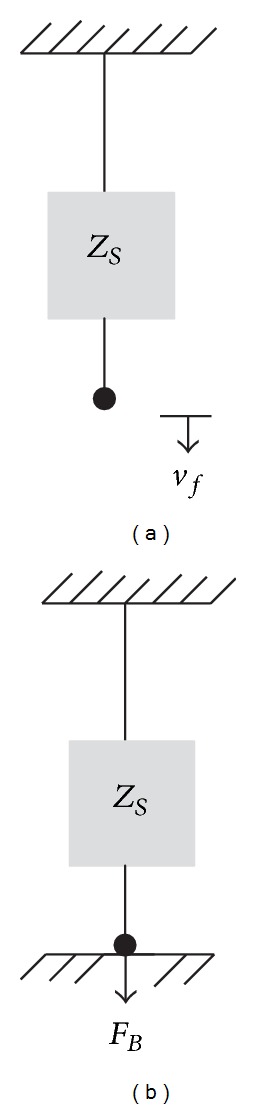
Free velocity and blocked force.

Consider a vibrating source with impedance *Z*_*S*_ freely suspended and vibrates with velocity *v*_*f*_ as shown in Figure 1(a). Without the presence of load or receiver structure to be attached, the velocity is called the “free velocity.” If the source is then attached rigidly on a rigid surface as in Figure 1(b), the injected force *F*_*B*_ by the source is called the “blocked force”. From definition

(1) $$\begin{array}{c} F_{B}=Z_{S}v_{f};\;\;F_{B}=\frac{v_{f}}{Y_{S}}, \end{array}$$

where *Y*_*S*_ = *Z*_*S*_^−1^ is the mobility of the source.

**Figure 2:**
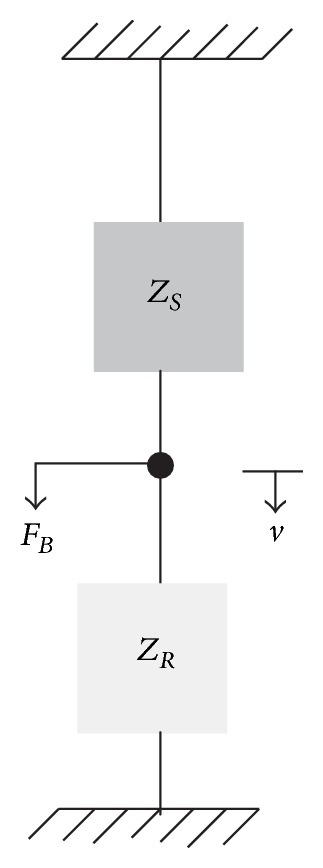
A source connected to a receiver.

Consider now the source is rigidly connected to a receiver structure with impedance *Z*_*R*_ as shown in Figure 2 and because of the rigid connection assumption, both the source and receiver move in the same velocity *v*. The blocked force at the contact point is now the sum of the force from the source *F*_*S*_ and that applied to the receiver *F*_*R*_ [8]. The blocked force can thus be written as

(2) $$\begin{array}{c} F_{B}=F_{S}+F_{R}=\left(Z_{S}+Z_{R}\right)v. \end{array}$$

Equation (2) can be rearranged to obtain the velocity at the contact point in terms of the source and receiver mobilities as well as the free velocity expressed as

(3) $$\begin{array}{c} v=\frac{F_{B}}{\left(Z_{S}+Z_{R}\right)}=\frac{v_{f}Y_{S}^{-1}}{1/Y_{R}+1/Y_{S}}=Y_{R}{\left(Y_{S}+Y_{R}\right)}^{-1}v_{f}. \end{array}$$

The vibration input power injected into the receiver is given by

(4) Pin=12Re{FR∗v}=12Re{ZR}v2,

where F* is the complex conjugate of *F*.

Assuming that the source is now attached to the receiver through *N* contact points, the formulation can be represented in terms of vectors and matrices given by

(5) Pin=12Re{FRHv}=12Re{vHZRHv},

where **F**_*R*_ and **v** are column vectors of size *N* × 1 and the impedance **Z** is a *N* × *N* matrix. The superscript *H* denotes the conjugate transpose. By substituting (3) into (5), the input power can be expressed as

(6) Pin=12Re{vfH[YS+YR]−HYR[YS+YR]−1vf}.

In terms of the blocked force, (6) can be rewritten as

(7) Pin=12Re{FBH[ZS+ZR]−HZR[ZS+ZR]−1FB}.

Consider now two extreme cases for idealisation where either the source has very high or very low mobility compared with that of the receiver. If the source has very low mobility so that |*Y*_*S*_| ≪ |*Y*_*R*_|, (6) reduces to

(8) Pin=12Re{vfH[YR]−Hvf}.

An example of this condition is a solid massive vibrating machine attached on a flexural floor. This type of source behaves as a “velocity source” which means that the velocity input to the receiver is insensitive to the dynamic behaviour of the receiver [9].

The second condition is where the mobility of the source is much higher than that of the receiver, so that |*Y*_*S*_| ≫ |*Y*_*R*_| or |*Z*_*S*_| ≪ |*Z*_*R*_|. Thus, (7) becomes

(9) Pin=12Re{FBH[ZR]−1FB}=12Re{FBHYRFB}.

This can be found, for example, in flexible vibrating machine mounted on a very thick floor. This source behaves as a “force source” which means that the force injected to the receiver is insensitive to the dynamic behaviour of the receiver structure [9].

### 2.2. Mobility Simplification

From (9) and (8), the mobility matrix **Y**_*R*_ for *N* contacts involves six components of excitations, that is, three translational and three rotational where 6*N* × 6*N* matrix size is therefore required. However, to simplify the problem, only translational force perpendicular to the receiver is taken into account. The matrix size reduces to *N* × *N* given by

(10) $$\begin{array}{c} Y=\left[\begin{array}{cccc} Y_{11} & Y_{12} & \cdots & Y_{1N}\\ Y_{21} & Y_{22} & \cdots & \cdots \\ \vdots & & \ddots & \vdots \\ Y_{N1} & \cdots & \cdots & Y_{NN} \end{array}\right], \end{array}$$

where *Y*_*ij*_ is the point mobility for *i* = *j* and transfer mobility for *i* ≠ *j*. At one point, the effect of other adjacent points can be represented by “collapsing” the point mobility and the transfer mobilities into a single mobility using the concept of effective mobility where for zero and random phase assumption between points, they are, respectively, expressed as [10]

(11) $$\begin{array}{c} Y_{i}^{\Sigma }\approx Y_{ii}+\sum_{i\neq j}^{N}Y_{ij}, \end{array}$$

(12) $$\begin{array}{c} {\left|Y_{i}^{\Sigma }\right|}^{2}\approx {\left|Y_{ii}\right|}^{2}+\sum_{i\neq j}^{N}{\left|Y_{ij}\right|}^{2}. \end{array}$$

### 2.3. Reception Plate Power

The reception plate method is a technique where a vibrating source under normal operating condition is connected to a flat plate structure. The total structure-borne power of the source is equal to the power generated on the plate given as [7]

(13) $$\begin{array}{c} P_{\text{in}}^{\text{Total}}=\eta_{R}\omega m_{R}\langle v_{R}^{2}\rangle , \end{array}$$

where *m*_*R*_ is the total mass of the plate, *η*_*R*_ is the total damping loss factor of the plate, *ω* is the operating frequency, and 〈*v*_*R*_^2^〉 is the spatial average of mean-squared velocity.

For the case where the structure-borne sound source has much lower mobility than that of the reception plate, |*Y*_*S*_| ≪ |*Y*_*R*_|, employing the effective mobility in (11) or (12), (8) can therefore be written as

(14) PinTotal≈12∑iNRe(1YRiΣ)|vfi|2.

Assuming small variations of effective mobility among the contact points, (14) can be further simplified as

(15) PinTotal≈12Re(1YRΣ)∑iN|vfi|2,

where *Y*_*R*_^Σ^ is average effective mobility across all contact points. From the reception plate power in (13), thus

(16) ηRωmR〈vR2〉=12Re(1YRΣ)∑iN|vfi|2.

As seen in (16), the total squared free velocities ∑_*i*_^*N*^|*v*_*fi*_|^2^ can be obtained from the reception plate method.

For the case where the reception plate has much lower mobility than that of the structure-borne source, |*Y*_*R*_| ≪ |*Y*_*S*_| and again assuming small variations of the mobilities, (9) becomes

(17) PinTotal≈12Re(YRΣ)∑iN|Fbi|2.

From the blocked force in (1), where *F*_*b*_ = *v*_*f*_/*Y*_*S*_, thus in (19)

(18) $$\begin{array}{c} \sum_{i}^{N}{\left|F_{bi}\right|}^{2}=\sum_{i}^{N}\frac{{\left|v_{fi}\right|}^{2}}{{\left|Y_{Si}^{\Sigma }\right|}^{2}}, \end{array}$$

with *Y*_*Si*_^Σ^ being the effective mobility of the source at the *i*th contact ponint. Again using the reception plate power and assuming small variations of source mobility across the contact point give

(19) ηRωmR〈vR2〉=12Re(YRΣ)1|YSΣ|2∑iN|vfi|2.

From (19), the average source mobility *Y*_*Si*_^Σ^ of the structure-borne sound source can now be obtained by also using the results of the total squared free velocity ∑_*i*_^*N*^|*v*_*fi*_|^2^ measured from the high mobility reception plate in (16).

## 3. Reception Plate Experiment

This section presents the reception plate measurement conducted with high and low mobility plates. A small table electric fan motor was used as a structure-borne source mounted rigidly on four contact points and operated at normal speed condition.

### 3.1. High Mobility Reception Plate

**Figure 3:**
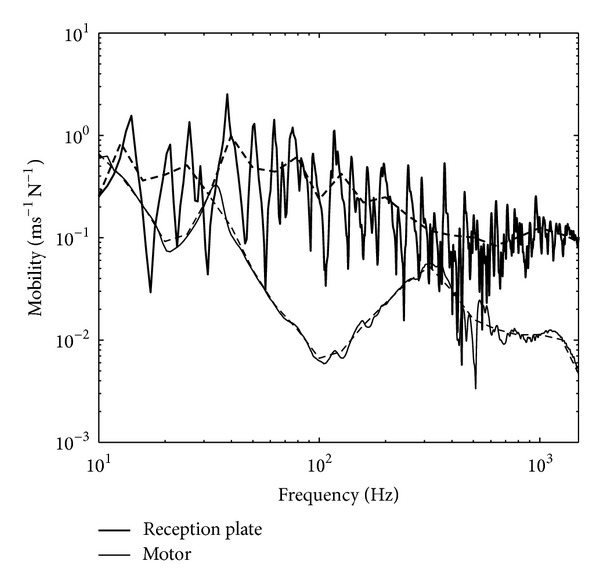
Comparison of measured average mobility from the high mobility reception plate and the fan motor: narrow band (solid line) and one-third octave band (dashed line).

The high mobility plate in the experiment used an aluminium plate having thickness of 1 mm and dimensions of 1.4 × 0.8 m. The plate was clamped with a steel frame and mounted on four rigid stands. Point mobilities were taken at four contact points (where the source was to be attached) using Kistler impact hammer Type 9722A500 and Kistler accelerometer Type 2021514. Mobilities at the motor feet were also taken.  Figure 3 shows the comparison of the averaged mobility between the plate and the motor where across the frequency particularly above 600 Hz, the mobility difference is 10 dB which supports the assumption used in (14).

**Figure 4:**
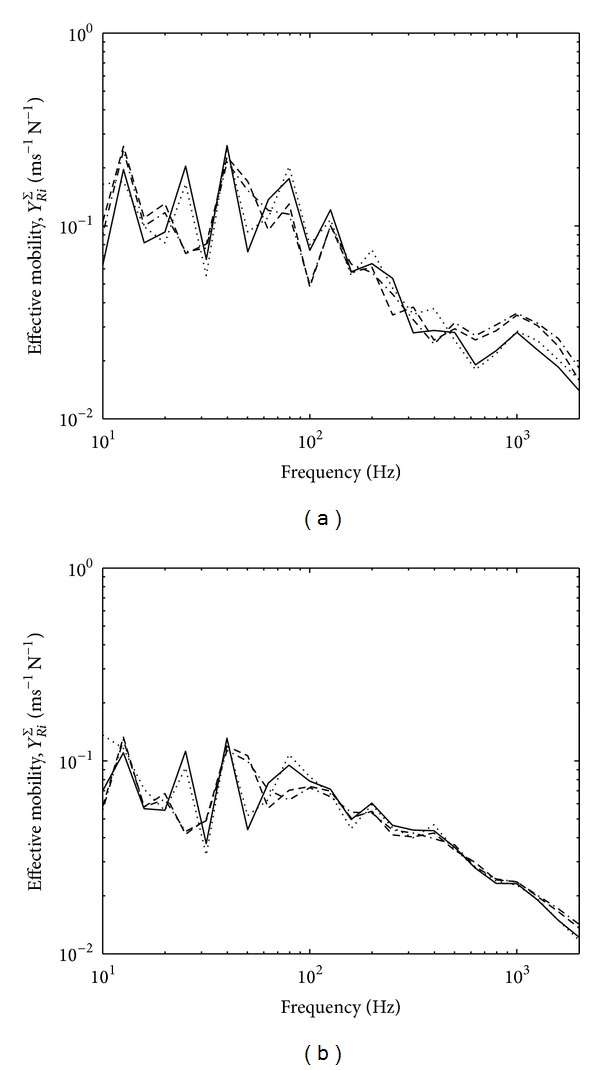
Effective mobility of the high mobility reception plate assuming: (a) zero phase and (b) random phase.

Figure 4 presents the variations of the effective mobilities for zero and random phase in one-third octave bands. The results for zero phase assumption from (11) as seen in Figure 4(a) show mobility variation within 1 dB which is acceptable for (15) to be valid. Much smaller variation can be seen for random phase assumption in Figure 4(b).

**Figure 5:**
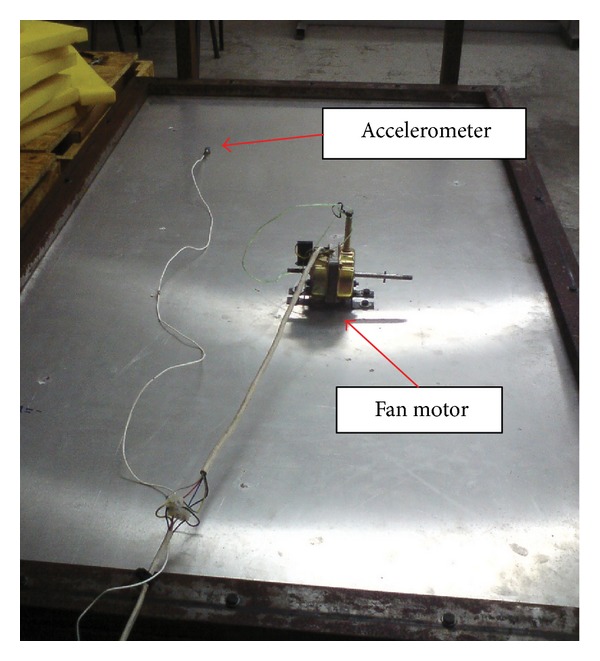
The fan motor attached on the high mobility reception plate.

**Figure 6:**
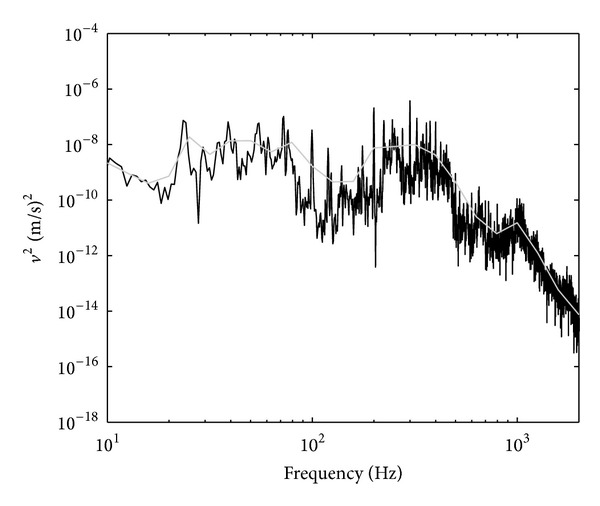
The measured spatially average mean-squared velocity of the high mobility reception plate (grey line: one-third octave band).

The motor was then attached on the plate positioned close, but off-centered on the plate to generate optimum modes of vibration. The arrangement can be seen in Figure 5. An accelerometer was attached at ten locations across the surface of the plate to measure the vibration velocity. The response location was carefully chosen so that the same point was not repeated due to symmetry. With the motor's normal operating speed, the spatially average mean-squared velocity 〈*v*_*R*_^2^〉 of the plate was measured. The result can be seen in Figure 6 where it can be observed that the response dominates below 1 kHz and rolls off above this frequency.

One more parameter to be measured before obtaining the free velocity from the reception plate method is the damping loss factor of the plate (see (13)). The damping factor can be measured in a reverberation chamber where it is inversely proportional to the measured reverberation time as conducted in [6]. However, the damping loss factor can also be measured conveniently using the input and spatially average squared mobility of the plate given by [7]

**Figure 7:**
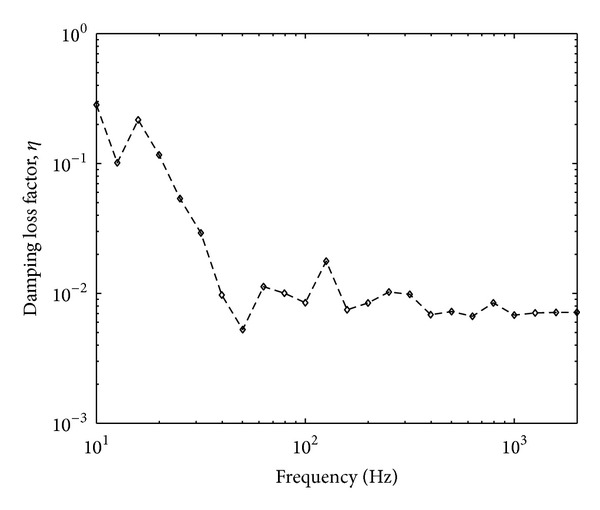
The damping loss factor of the high mobility reception plate.

(20) ηR=Re{Yp}ωm¨RSR〈|Yt|2〉,

where *Y*_*p*_ is the point mobility and 〈|*Y*_*t*_|^2^〉 is the spatially average squared transfer mobilities. Five measurement locations out of ten points for measuring the spatially average squared velocity were chosen for the measurement of input and transfer mobilities. The result of the measured damping loss factor is plotted in Figure 7 in one-third octave bands. Constant results can be seen above 200 Hz where the damping loss factor of the plate is around 0.007.

**Figure 8:**
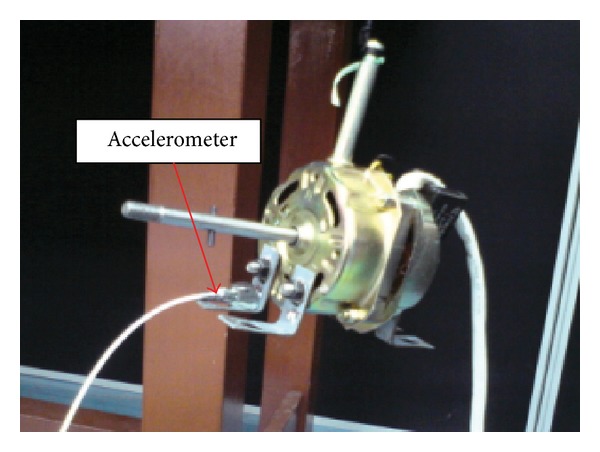
Measuring the free velocity directly at the feet of the fan motor running at normal speed.

Figure 8 shows the table fan electric motor used in the experiment with an accelerometer attached on one of its L-shape feet in order to measure the direct free velocity to be compared with that from the reception plate method.

**Figure 9:**
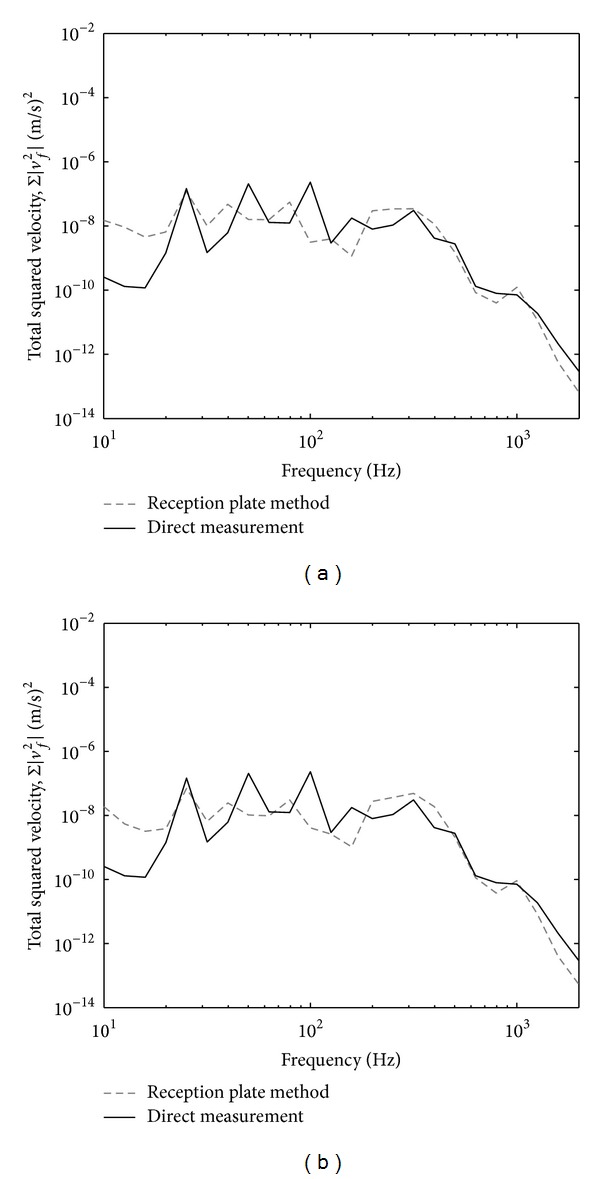
Comparison of the squared free velocity obtained from the reception plate method (thick line) and direct measurement (thin line): (a) zero phase and (b) random phase.

By using (19), the estimation of the total squared free velocity ∑_*i*_^*N*^|*v*_*fi*_|^2^ of the motor can be obtained and presented in Figure 9 compared with that from the direct measurement. It can be seen that the estimated squared free velocity follows the trend of that from the direct measurement consistently. The results can be seen to have good agreement above 200 Hz within 5 dB discrepancy for both zero phase and random phase assumptions of the effective mobility. Discrepancy of 10 dB can be seen at low frequency below 100 Hz due to small modal density of the reception plate on which the reception plate power in (13) is based. The L-shape feet of the motor might also give effect it these creates high stiffness structure where vibration power from the motor was not fully transmitted to the reception plate.

### 3.2. Low Mobility Reception Plate

**Figure 10:**
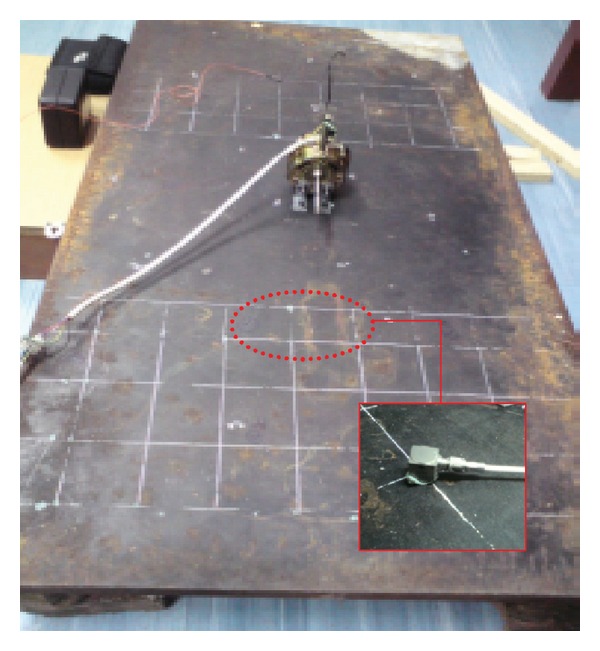
The electrical fan motor on the low mobility reception plate.

For the low mobility reception plate, a 2.5 cm thick steel plate was used with dimensionts of 1.24 × 0.61 m as seen in Figure 10. The plate was rested on the floor where rubber pads were located between the plate and the supported bricks to prevent unwanted reflected vibration waves and to let the plate to move in free-free edges.

Note that in (19) the input power now also depends on the mobility of the source. Due to high stiffness of the L-shape of the source feet, careful mobility measurement has therefore been taken by exciting the body of the motor as close as possible to the connection with the L-shape feet and mounted the accelerometer on each of the motor foot to record the vibration response.

**Figure 11:**
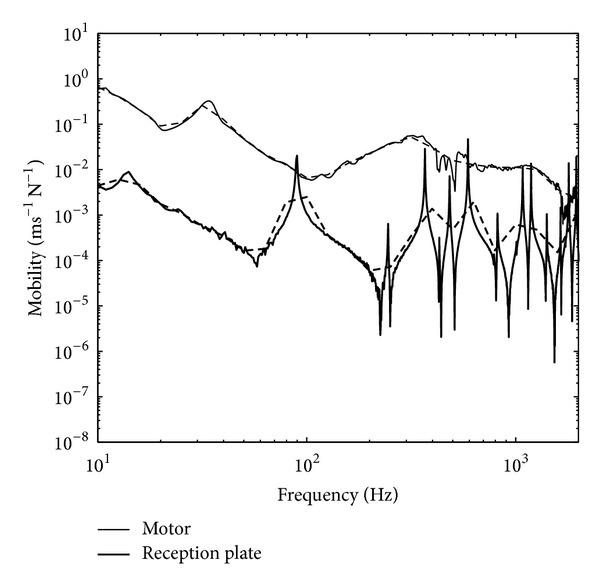
Comparison of measured average mobility from the low mobility reception plate and the fan motor: narrow band (solid-line) and one-third octave band (dashed-line).

Figure 11 plots the comparison between the measured average mobility of thick plate mobility and the motor. Distinct peaks can be clearly seen in the plate mobility indicating low modal density of the plate. This raises problem when applying (13) where diffuse field vibration is required. However, the level of the plate mobility can be seen to be more than 10 dB lower than the source mobility which fulfils the impedance mismatch assumption in (19).

**Figure 12:**
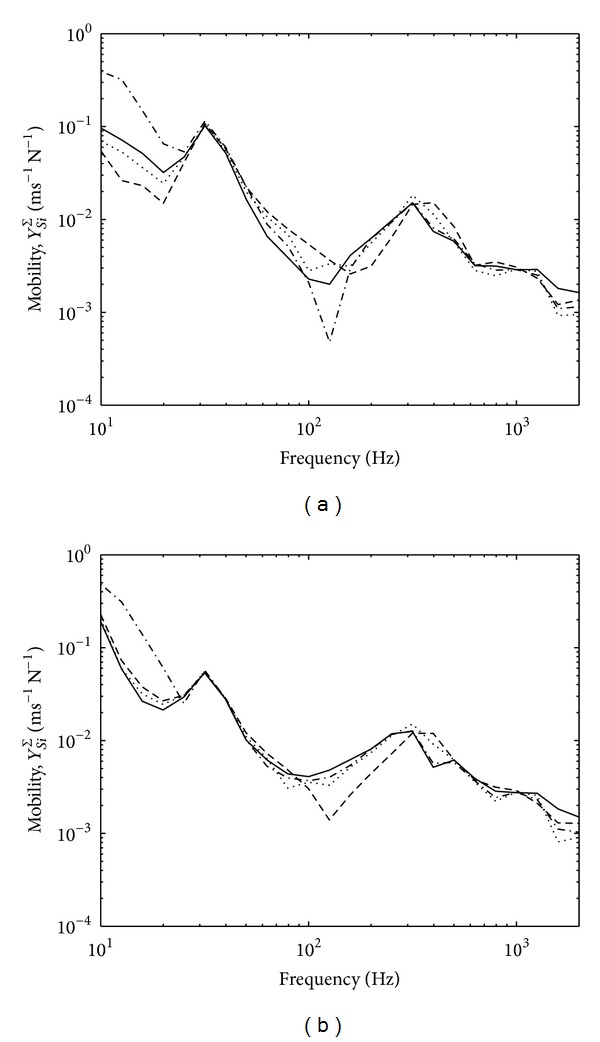
Effective mobility of source by assuming (a) zero phase and (b) random phase.

**Figure 13:**
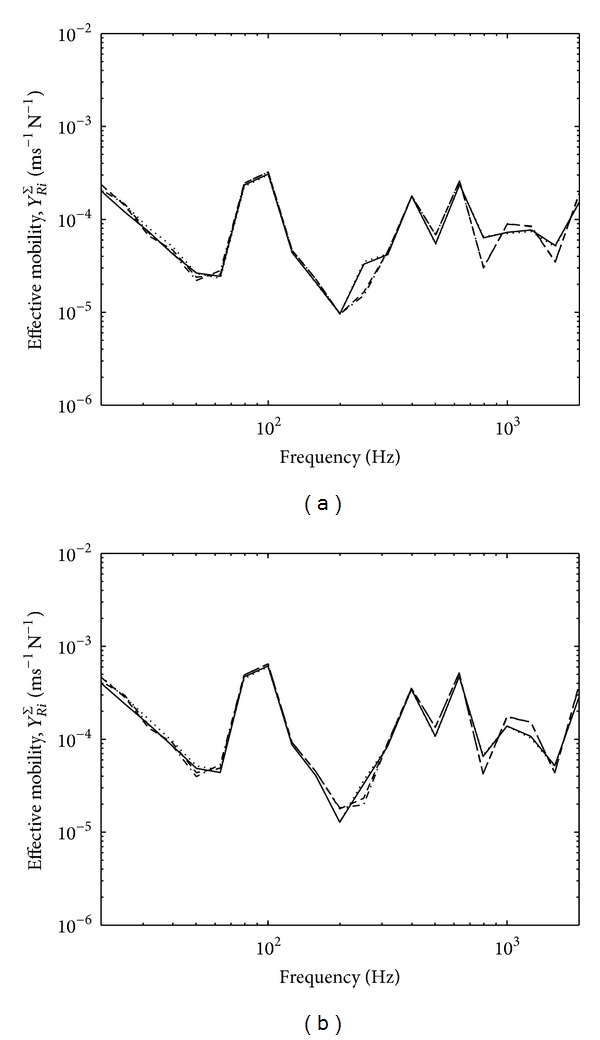
Effective mobility of the thick plate by assuming (a) zero phase and (b) random phase.

Equation (19) also assumes small variation of effective source mobility. This is shown in Figure 12 for zero and random phase assumptions in one-third octave bands. The variation can be seen to be less than 5 dB for the four contact points across the frequency range except at below 20 Hz and around 120 Hz which might be due to the nature of the corresponding foot. The variation of the effective mobility of the thick reception plate can also be seen in Figure 13 to be sufficiently small.

**Figure 14:**
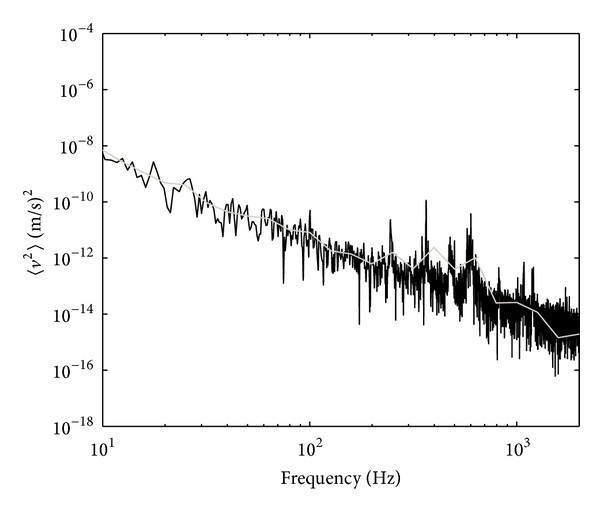
The measured spatially average mean-squared velocity of the low mobility reception plate (grey line: one-third octave band).

The spatially average of mean-squared velocity of the plate is shown in Figure 14. Due to small response at the locations away from the motor (e.g., near the plate edges), spatial averaging was only done for the measurement points close to the contact points. The response can be seen to decrease as the frequency increases.

**Figure 15:**
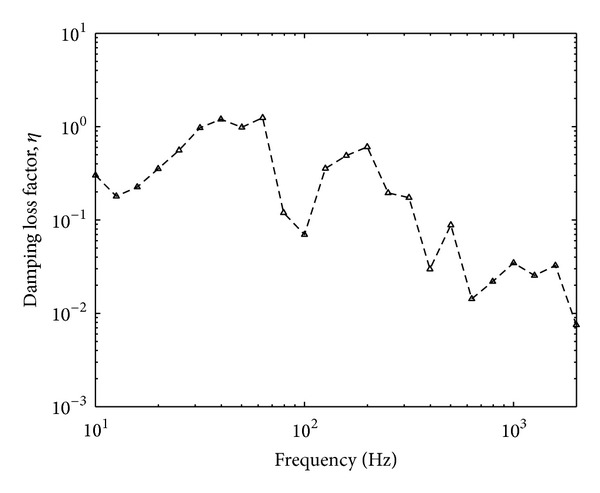
The damping loss factor of the low mobility reception plate.

The measured damping loss factor in Figure 15 also decreases with frequency due to low modal density. Constant level of loss factor values is expected as for the high mobility plate in Figure 7, which in this case, is expected to be at higher frequency above 2 kHz. The damping loss factor is therefore assumed similar to that of the thin plate, that is, 0.005 which is still a reasonable value for a plate without damping treatment.

**Figure 16:**
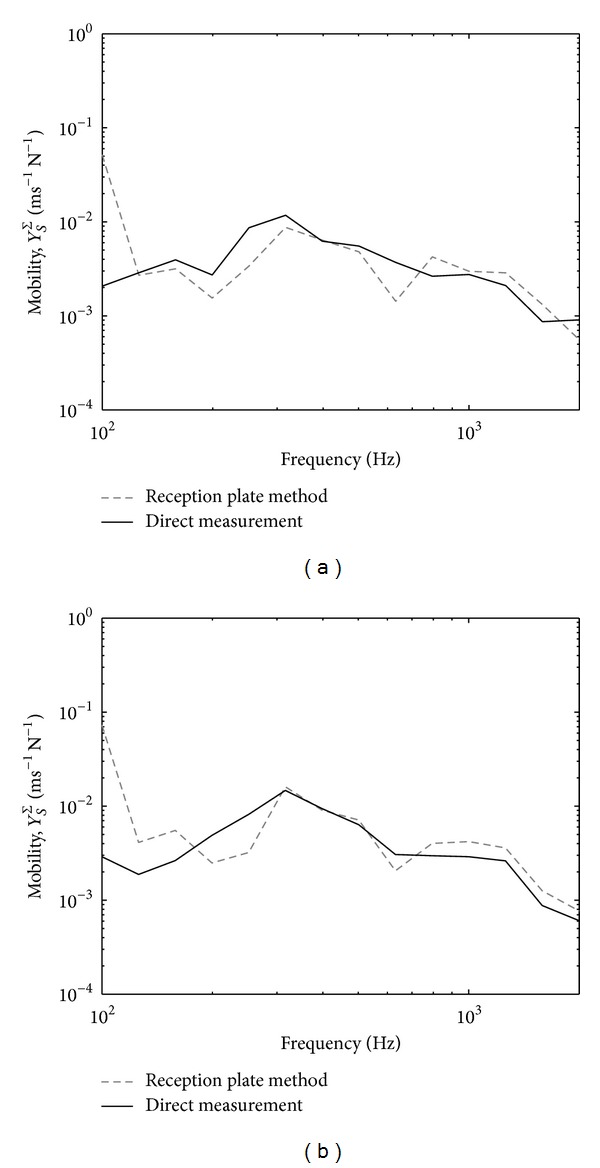
Comparison of the squared free velocity obtained by the reception plate method (thick line) and direct measurement (thin line): (a) zero phase and (b) random phase.

Finally, the results for the estimation of the effective source mobility are presented in Figure 16. Again good agreement with the direct measurement can be seen above 200 Hz where the reception plate result follows the trend of the direct measurement, as also obtained for the total free velocity in the thin reception plate (see Figure 9). Large discrepancy occurs below 200 Hz which is due to the low modal density of the reception plate.

## 4. Conclusion

Characterisation of a structure-borne source of a table fan motor using the reception plate method has been done successfully. Good agreement of the estimated free velocity and mobility of the source has been achieved from mid to high frequency. However, the use of a low mobility plate to estimate the source mobility is found to be cumbersome as it has low modal density, which is contrary to the condition required by the reception plate power. Careful measurement of the vibration velocity is therefore important to consider the modal behaviour of the plate especially at the area close the excitation point. For the future work, instead of the mean-average result, possible range of the data results of the free velocity and mobility from the measured structure-borne source in terms of its statistical variation across the frequency is also of interest to account the uncertainty coming from source and the receiver.
